# Unique treatment principles in the service of speech treatment – The VML method

**DOI:** 10.1192/j.eurpsy.2022.2274

**Published:** 2022-09-01

**Authors:** E. Vashdi

**Affiliations:** Yaelcenter institute, Clinic, Aloney Aba, Israel

**Keywords:** Apraxia of speech, autism, VML method, Teaching principles

## Abstract

**Introduction:**

Childhood Apraxia of Speech (CAS) was declared as a motor speech disorder by ASHA (2007). The prevalence of CAS is yet to determined due to difficulties in reliable diagnosis, however 63.6% of children with ASD were found to Apraxic (Tierney et al, 2015), hence prevalence would be at least 1/100 (ASD prevalence is 1/54).

**Objectives:**

The consequences of inability to speak in early and advanced developmental age are many and might include frustration, anger, depression, communication deficit, language delay, poor social skills and behavioural problems. Not only treating motor speech disorder is challenging enough, the external consequences make it far more difficult.

**Methods:**

The VML method (Verbal Motor Learning) was designed to treat the motor aspect of speech. It uses manual techniques and motor learning principles in order to directly teach the basic milestones of speech for children with CAS. In addition, the importance of the manner of the practice was established over the years, extracting 16 unique treatment principles. These principles guide the therapist regarding HOW to perform the practice, while giving an answer to the external CAS consequences.

**Results:**

The principles are the platform, managing energy levels, relationship, attention, goal-oriented thinking, emotional safety, treatment structure, rhythm, timing, threshold point, support, dynamic thinking, ecological treatment, proactivity, clarity and commitment.

**Conclusions:**

The lecture will introduce the 16 principles briefly, and demonstrate the use of them via treatment videos.

**Disclosure:**

I am the founder of the VML method and I teach it in various countries.

